# A TNFR2-Agonist Facilitates High Purity Expansion of Human Low Purity Treg Cells

**DOI:** 10.1371/journal.pone.0156311

**Published:** 2016-05-25

**Authors:** Xuehui He, Sija Landman, Stijn C. G. Bauland, Juliette van den Dolder, Hans J. P. M. Koenen, Irma Joosten

**Affiliations:** 1 Laboratory of Medical Immunology, Department of Laboratory Medicine, Radboud University Medical Center, Nijmegen, The Netherlands; 2 Sanavisie Bodyclinic, Mill, The Netherlands; 3 Hycult Biotech, Uden, The Netherlands; Sorbonne Universités, FRANCE

## Abstract

Regulatory T cells (Treg) are important for immune homeostasis and are considered of great interest for immunotherapy. The paucity of Treg numbers requires the need for *ex vivo* expansion. Although therapeutic Treg flow-sorting is feasible, most centers aiming at Treg-based therapy focus on magnetic bead isolation of CD4+CD25+ Treg using a good manufacturing practice compliant closed system that achieves lower levels of cell purity. Polyclonal Treg expansion protocols commonly use anti-CD3 plus anti-CD28 monoclonal antibody (mAb) stimulation in the presence of rhIL-2, with or without rapamycin. However, the resultant Treg population is often heterogeneous and pro-inflammatory cytokines like IFNγ and IL-17A can be produced. Hence, it is crucial to search for expansion protocols that not only maximize *ex vivo* Treg proliferative rates, but also maintain Treg stability and preserve their suppressive function. Here, we show that *ex vivo* expansion of low purity magnetic bead isolated Treg in the presence of a TNFR2 agonist mAb (TNFR2-agonist) together with rapamycin, results in a homogenous stable suppressive Treg population that expresses FOXP3 and Helios, shows low expression of CD127 and hypo-methylation of the *FOXP3* gene. These cells reveal a low IL-17A and IFNγ producing potential and hardly express the chemokine receptors CCR6, CCR7 and CXCR3. Restimulation of cells in a pro-inflammatory environment did not break the stability of this Treg population. In a preclinical humanized mouse model, the TNFR2-agonist plus rapamycin expanded Treg suppressed inflammation *in vivo*. Importantly, this Treg expansion protocol enables the use of less pure, but more easily obtainable cell fractions, as similar outcomes were observed using either FACS-sorted or MACS-isolated Treg. Therefore, this protocol is of great interest for the *ex vivo* expansion of Treg for clinical immunotherapy.

## Introduction

Following identification of Treg, the immunomodulating role of Treg was demonstrated in a variety of preclinical autoimmunity and transplantation models. Their clinical relevance was highlighted by demonstrating that the immunosuppressive function of Treg was hampered in autoimmunity and allergy. Clinical application of Treg has been hampered by the paucity of Treg cell numbers and the fact that standard methods of *ex vivo* Treg expansion produce heterogeneous cell populations [1]. For clinical application of Treg-based immunotherapy isolation of Treg using a good manufacturing practice (GMP) system is required. Clinical grade flow-sorting which retrieves highly pure Treg is restricted to a few clinic centers worldwide. In contrast, magnetic bead isolation of CD4+CD25+ Treg using a GMP compliant closed system, such as CliniMACS, that results in lower Treg purity [2] is more generally used. For Treg expansion most centers apply polyclonal expansion protocols making use of anti-CD3 plus anti-CD28 mAb stimulation in the presence of rhIL-2 together with or without rapamycin [2–8]. This results in a heterogeneous Treg population revealing inadvertent pro-inflammatory (IL-17A, IFNγ) cytokine producing potential [9]. The fact that human Treg could lose FOXP3 expression and suppressive functions and acquire the capacity to produce pro-inflammatory cytokines under pro-inflammatory micro-environmental conditions [10, 11] might have important implication for Treg-based clinical therapy. Therefore, it is essential to develop highly efficacious expansion protocols that promote strong Treg proliferation whilst maintaining or promoting Treg stability and suppressor function. We and others have evidence that pharmaceutical agents influence Treg phenotype and functional capacity [12–14], indicating that by delicate selection of pharmaceutical agents it is possible to further support the stability of human Treg. In this respect, the mTOR inhibition by rapamycin is an interesting example, since it has been shown to promote preferential outgrowth of highly suppressive Treg [4, 14, 15]. In contrast to effector T cells (Teff), Treg are less sensitive to mTOR inhibition by rapamycin since Treg proliferation and survival preferentially depends more on the STAT5 [16] and Pim kinase pathways [17].

Tumour necrosis factor receptor 2 (TNFR2) expression, in contrast to TNFR1, is restricted to lymphocytes and mainly binds membrane bound TNF instead of soluble TNF [18]. The binding of TNFα to TNFR2 provides costimulatory signals to T cells that enhance T cell proliferation and cell survival [19]. TNFR2 signalling is important for Treg, as TNFR2 deficient mice had reduced numbers of thymic and peripheral Treg [20], and TNFR2 ^-/-^ Treg were not able to control inflammatory responses *in vivo* [21]. Human Treg also express a higher level of TNFR2 than Teff [22, 23], and TNFR2+ Treg exhibited the most potent suppressive capacity [24]. The interaction of TNF-TNFR2 promotes Treg proliferation and survival via the activation of the NFκB pathway [25]. The fact that a TNFR2-agonist drives human Treg into a homogeneous population with potent suppressive capacity [22] indicates that TNFR2 is a valuable target for facilitating *ex vivo* expansion of human Treg. In this study, we show that expansion of low purity MACS-isolated human Treg in the presence of TNFR2-agonist and rapamycin results in a stable homogenous FOXP3+, Helios+, CD127^low^ Treg population that shows profound suppressor potential both *in vitro*, and *in vivo* in a preclinical humanized mouse model. Irrespective of the purity of Treg at the start of cell culture, i.e. either low purity MACS-isolated or high purity FACS-sorted Treg, cells expanded in the presence of TNFR2-agonist plus rapamycin, showed a stable Treg phenotype and potent suppressive capacity. Re-stimulation of cells in a pro-inflammatory environment did not break the stability of this Treg population. Thus, a TNFR2-agonist based expansion protocol shows great potential for *ex vivo* Treg expansion for clinical purposes.

## Materials and Methods

### Isolation of Treg

Peripheral blood mononuclear cells (PBMCs) were isolated by density gradient centrifugation (Lymphoprep, Nycomed Pharma AS, Oslo, Norway) of buffy coats obtained from healthy blood donors (Sanquin Blood Bank, Region South-East, Netherlands) upon written informed consent, according to the Dutch law. CD4+ T cells were enriched using the RosetteSep^TM^ human CD4+ T cell enrichment cocktail and processed according to manufacturer’s recommendations (StemCell Technologies, Vancouver, Canada). This resulted in a >95% purified CD4+ T cells and the absence of CD8+ cells. To obtain high purity Treg, FACS sorting of CD4+CD25^high^ Treg was performed using a BD FACSAria cell sorter (BD Biosciences, Erembodegem, Belgium) after labeling CD4+ cells with CD25/Pe-Cy7(M-A251; BD Biosciences), termed as FACS-sorted Treg. More than 97% of Treg were FOXP3+ after cell sorting. Less pure MACS-isolated CD4+CD25+ Treg were prepared using human CD25 microbeads (Miltenyi Biotech, Bergisch Gladbach, Germany), according to manufacturer’s instructions. To mimic the purity of clinic grade isolation, 15–20 μL of CD25 microbeads for every ten million CD4+ cells were used. The resultant Treg were 60–80% positive for FOXP3.

### Flow cytometry

Cells were phenotypically analyzed using a multicolor flow cytometer Navios (Beckman-Coulter, Mijdrecht, Netherlands). The following conjugated mAb were used: CD127(R34.34)/APC-AF700, CD25(M-251)/APC or /Pe-Cy7 (BD), CD27(1A4-CD27)/PE-Cy5.5, CD3 (UCHT1)/ECD, CD4(1388.2)/PE-Cy5.5, CD62L(DREG56)/ECD, HLA-DR(Immun-357)/FITC, CD8(B9.11)/APC-AF700, (all from Beckman-Coulter), CCR6(11A9)/Biotin (BD Bioscience), CCR7(150503)/PE (R&D, Minneapolis, US), CXCR3(G025H7)/APC-Cy7 (Biolegend, San Diego, US), TNFR2(MR2-1)/FITC (Hycult, Uden, Netherlands), TNFR2 (#22235) /APC (R&D, Minneapolis, US), and Fix-viable-Dye labeled with APC-eFluo780 (eBioscience, Vienna, Austria). For intracellular staining, FOXP3 (PCH101) /eFluo660 and Helios(22F6)/AlexFluo647 (both from eBioscience) were used after fix-perm-treatment of cells, according to the manufacturer’s instructions. For intracellular cytokine staining, cells were stimulated with phorbolmyristate acetate (PMA, 12.5 ng/mL), ionomycin (500 ng/mL) and brefeldin A (5 μg/mL) for 4 hours before starting of FACS staining. IFNγ(45.B3)/Pe-Cy7 and IL-17A (eBio64DEC1) /Alexa488, (both from BD Bioscience) were used. Isotype matched antibodies were used to define marker settings. Data were analyzed using the software Kaluza (Beckman-Coulter).

### Protocols used for *ex vivo* Treg expansion

1. High purity FACS-sorted Treg (5 x 10^4^) were stimulated with anti-CD3/anti-CD28 mAb-coated microbeads (Cat. no. 11131D, Invitrogen, Bleiswijk, Netherlands) in a 1:2 bead-to-cell ratio and exogenous rhIL-2 (200 U/mL, Proleukine, Amsterdam, Netherlands). TNFR2-agonist (2.5 μg/mL, mAb MR2-1, Hycult) and/or rapamycin (1μM, Sigma-Aldrich, Zwijndrecht, Netherlands) was added at the start of the cultures. Cells were harvested and analyzed at day 7 as described.
2. Low purity MACS-isolated Treg (5 x 10^4^) were stimulated with anti-CD3/anti-CD28 mAb-coated microbeads (Cat. no. 11131D, Invitrogen) in a 1:2 bead-to-cell ratio. TNFR2-agonist (2.5 μg/mL, Hycult) and/or rapamycin (1μM) was added at the start of the cultures. On day 2, exogenous rhIL-2 (750 U/mL) was added to the culture. Every 2 or 3 days the culture medium was replenished by fresh culture medium containing rapamycin (1μM) (until day 7) and 750 U/mL rhIL-2. On day 9, additional TNFR2-agonist (2.5 μg/mL) was supplemented. On day 16, cells were harvested and analyzed.

### Co-culture suppression assays

The suppressor capacity of expanded Treg was studied using co-culture suppression assays. Treg were expanded for 7 (FACS-sorted Treg) or 16 (MACS-isolated Treg) days under the conditions described. Thereafter, Treg were collected, washed and added at different ratio’s to CFSE-labeled CD4+CD25- responder T cells (Tresp) together with anti-CD3/anti-CD28 mAb-coated beads (1:5 bead-to-cell ratio) for 3 days. Proliferation of Tresp was determined by analyzing CFSE dilution as described previously [26].

### *FOXP3* gene methylation

The *FOXP3* methylation status was analyzed by bisulphate sequencing as described previously [27]. In brief, geneGenomic DNA was isolated from either MACS-isolated CD4+CD25+ Treg or expanded Treg under each treatment group using the QIAamp DNABloodMini kit (Qiagen, Venlo, Netherlands), Bisulfite converted and amplified using bisulfite-specific polymerase chain reaction (PCR) (forward 59 TGGATATTTGGTTAGAGT TAAGAAT 39 and reverse 59 ACCTAACACTCTCAAAACTTCAAAC 39). The purified PCR product was sequenced on an ABI 3130 Genetic Analyzer (Applied Biosystems, Bleiswijk, Netherlands), and analyzed using Sequencing Analysis version 5.4 software (Applied Biosystems).

### Humanized skin inflammation mouse model

The humanized skin inflammation mouse model used in this study has been described previously [28]. In brief, human abdominal skin from healthy individuals obtained after elective surgery (Sannavisie Bodyclinic, Mill, Netherlands) was transplanted onto 6–8 week old female B17.B6-Prkdc^scid^Lyst^bg^/Crl (SCID/beige) mice, and allowed to engraft for 3 weeks. Next, mice were intra peritoneally (i.p) injected with 10–40 x 10^6^ huPBMC in the absence or presence of rapamycin expanded Treg (^Rap^Treg) or Rapamycin plus TNFR2-agonist expanded Treg (^R/T^Treg) at a ratio huPBMC: Treg of 1:1 or 1:2. Mice were sacrificed 3 weeks after the injection of the human immune cells. Tissues of interest were collected, and the histological analysis of the grafts was performed thereafter.

All the animal experimental procedures were in accordance with the international welfare guidelines taking into consideration of the 3Rs (Refinement, Reduction, and Replacement) and approved by the institutional ethical animal care committee of the Radboud University Nijmegen (Number DEC2013-023). Mice were sacrificed using the orbita extraction under anesthesia followed by cervical dislocation. The use of human skin and peripheral blood were approved and in accordance with the regulations set by the Medical Ethical Committees for human research of the Radboudumc. Human skin (from elective surgery) and buffy coats were from healthy donors, who gave written informed consent for scientific use. Buffy coats were purchased from Sanquin Blood Bank, Nijmegen, Netherlands.

### Histology & Immunohistochemistry

Human skin grafts were fixed in neutral buffered 4% formalin (Mallinckrodt Baker, Inc Deventer, Netherlands) for 4 hours, processed and embedded in paraffin. Then, 6 μm sections were cut and the slides were stained with Hematoxylin-Eosin (HE) or processed for immunohistochemical staining. Human CD3 mAb (clone7.2.38, Abcam, Cambridge, UK) was used to stain human CD3+ T cells. Antibody stainings were visualized using the Dako Cytomation EnVision+system-HRP (ABC) kit (DAKO, Glostrup, Copenhagen, Denmark) combined with 3,3’-diaminobenzidine tetrahydrochloride (DAB, brown, Sigma-Aldrich). Sections were photographed using a microscope (Axiokop2 MOT; Zeiss, Sliedrecht, Netherlands), digital camera (Axiocam MRc5; Zeiss) and AxioVision software (Zeiss).

### Determination of epidermal thickness

Histologic assessment of the grafts was performed using the light microscopy after transplantation of human skin. The mean epidermal thickness was calculated using the program Visiopharm Integrator System (VIS) (Visiopharm, Hørsholm, Denmark) as epidermal area divided by epidermal surface length.

### Image analysis of immunohistochemistry

To enumerate human CD3+ T cells, representative pictures were made at 20× magnification. A representative region of interest (ROI) was drawn from the lowest epidermal papilla till 300 mm depth into the dermis. Cell quantification was performed by setting a threshold and relating this to a number of cells per mm^2^. For evaluation of number of CD3+ cells, positively stained cells were counted manually in CD3 infiltrated areas of the tissues and the number was reported per mm^2^.

### Statistics

Statistical analysis was performed using the GraphPad Prism software version 5.0 (GraphPAd Software Inc., San Diego, US). For comparison between two groups, a Wilcoxon paired t-Test was used. For comparison among multiple groups, a Kruskal-Wallis test plus Dunns post hoc test or Friedman test plus Dunns post hoc test was used, where appropriate. P values of <0.05 were considered significant.

## Results

### Expansion of high purity FACS-sorted Treg in the presence of TNFR2-agonist and rapamycin preserves Treg suppressor function and stability

**Fig 1:**
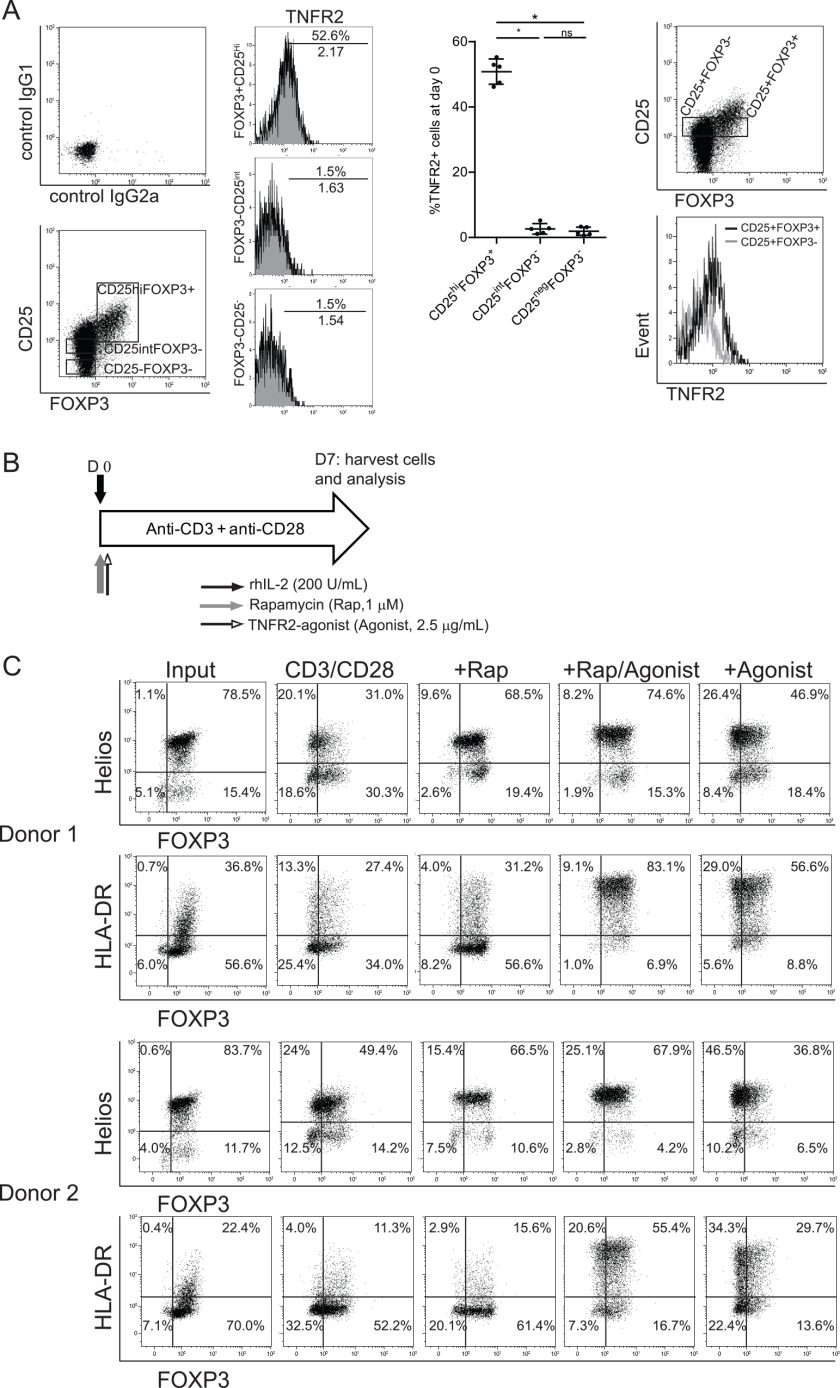
TNFR2-agonist preserves the expression of FOXP3 and enhances Helios and HLA-DR, markers associated with highly suppressive function. (A) Flow cytometry analysis of human CD4+ cells immediately after cell preparation. Left panel: Histograms show the expression of TNFR2 within CD25^high^FOXP3+, CD25^int^FOXP3-, and CD25^neg^FOXP3- subsets. A representative experiment out of five is shown. Numbers of %TNFR2+ cells (top) and mean fluorescence intensity of TNFR2 (low) are listed in the histograms. Middle panel: Cumulative data showing the percentage of TNFR2+ cells within different cell subsets as shown on the X-axis (N = 5). Right panel: Example overlay histogram showing the expression of TNFR2 within CD25+FOXP3+ and CD25+FOXP3- subsets. A Friedman with Dunns post hoc test was used for comparison among groups. Asterisks indicate significant differences. (B) Schematic overview of expansion strategy for FACS-sorted Treg, as described in Materials and Methods. (C) Flow cytometry of high purity FACS-sorted Treg before (input) and after cell expansion under the indicated conditions. Dot plots showing surface expression of HLA-DR, and intracellular expression of FOXP3 and Helios. Numbers within the gated regions show the percentage of positive cells. Data derived from two different healthy donors are shown. Rap: rapamycin; Agonist: TNFR2-agonist.

**Fig 2:**
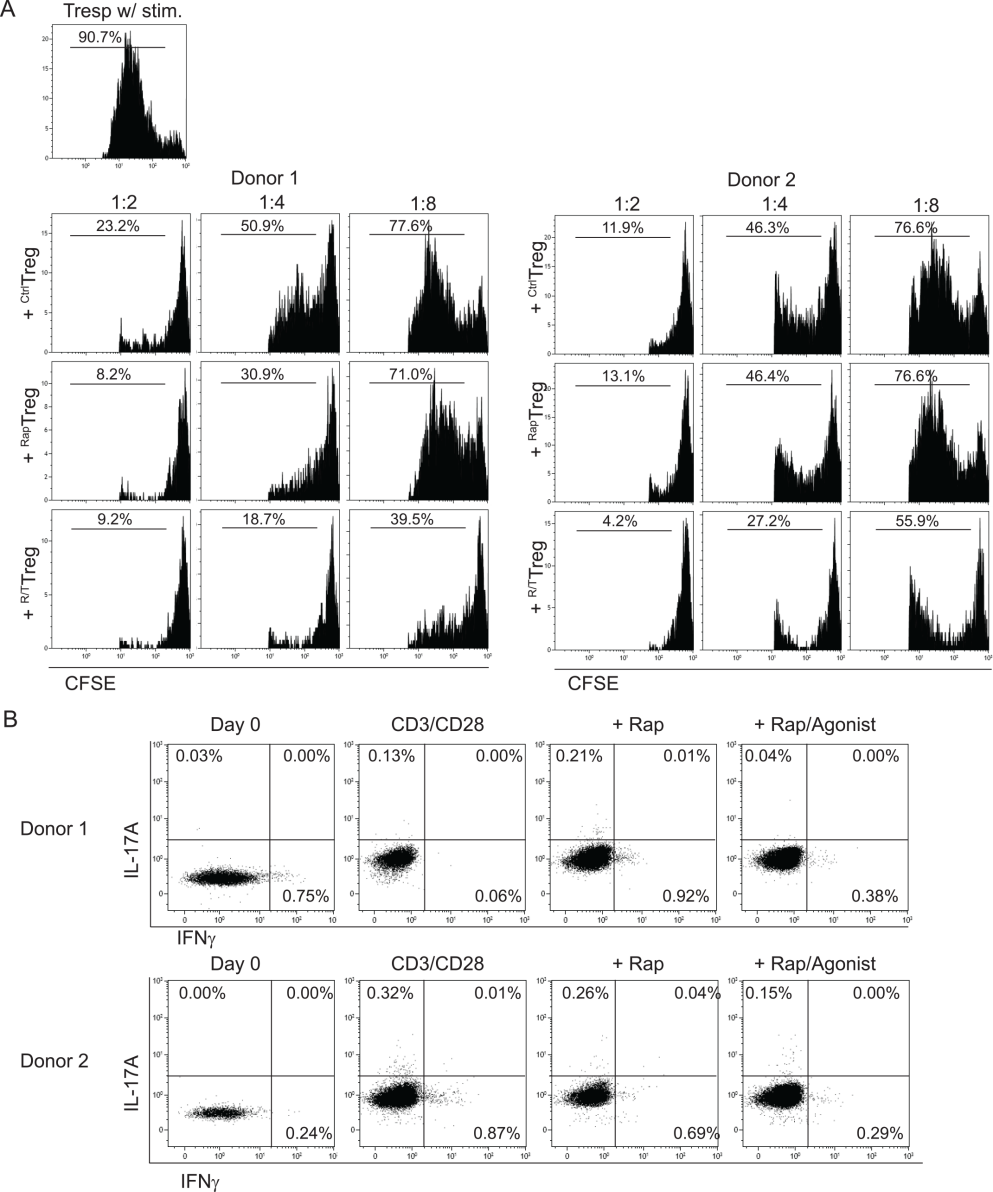
TNFR2-agonist plus rapamycin expanded FACS-sorted Treg reveal a high suppressive capacity and less IL-17A and IFNγ producing potential. High purity FACS-sorted human Treg were stimulated as described in Material and Methods. At day 7 of the cell cultures, expanded Treg were harvested, washed, and analyzed for their suppressive capacity in a CFSE-based co-culture suppression assay. ^Ctrl^Treg, ^Rap^Treg and ^R/T^Treg represent cells expanded in the absence or presence of rapamycin-only or TNFR2-agonist plus rapamycin, respectively. (A) Histograms showing the inhibition of proliferation of responder cells following the addition of graded doses of Treg. The ratio of Treg:Tconv is indicated on the top. Numbers show the percentage of divided cells. (B) Flow cytometry of intracellular IL-17A and IFNγ of Treg before (day 0) and after expansion under the indicated conditions. Data derived from two different healthy donors are shown. Numbers show the percentage of positive cells. Rap: rapamycin; Agonist: TNFR2-agonist.

We began by examining the expression of TNFR2 on human CD4+ T cells. Based on the expression of CD25 and FOXP3, CD4+ T cells were gated as CD25^high^FOXP3^+^, CD25^int^FOXP3^-^, and CD25^neg^FOXP3^-^ subsets. The CD25^high^FOXP3^+^ subset showed the highest expression levels of TNFR2. High expression of TNFR2 on Treg was further confirmed by showing that in contrast to the high numbers of positive cells in the CD25+FOXP3+ population, only few CD25+FOXP3- cells expressed this receptor (Fig 1A). Next, we studied the effect of additional TNFR2-agonist [22] stimulation on suppressor function and stability of highly purified Treg. To this end, high purity FACS-sorted human CD4+CD25^high^ Treg were stimulated using anti-CD3/CD28-mAb coated beads and rhIL-2, in the presence or absence of TNFR2-agonist and/or rapamycin, and cultured for a week (Fig 1B). Purity of the Treg population based on FOXP3 expression was 93.5% ± 3 (Mean ± SD), and 80.1% ± 2.5 (Mean ± SD) of these cells co-expressed Helios, which is in line with the literature [29] that states that Helios+ and Helios- subsets co-exist within human FOXP3+ Treg. Upon stimulation with CD3/CD28 mAb-coated microbeads in the presence of 200U/ml recombinant human IL-2, a percentage of Treg lost the expression of FOXP3, whereas the presence of rapamycin-only or TNFR2-agonist plus rapamycin helped Treg maintain FOXP3 and enhanced Helios expression. Interestingly, the expression of HLA-DR, a molecule associated with potent Treg suppressive function [30], was highly enhanced by the use of TNFR2-agonist (Fig 1C). With regard to chemokine receptor expression, CCR5 was hardly expressed on Treg, regardless of the condition tested. Combined use of TNFR2-agonist plus rapamycin down-regulated CCR6 and CXCR3 expression, but hardly affected CCR7 as compared to CD3/CD28 stimulation in the presence or absence of rapamycin (S1 Fig),

An important feature of Treg is their suppressive capacity. We assessed this functional capacity in a CFSE-based co-culture suppression assay, using autologous CD4+CD25- T cells as responder cells. Treg expanded with TNFR2-agonist plus rapamycin (^R/T^Treg) showed enhanced suppressor capacity as compared to Treg expanded in the absence (^Ctrl^Treg) or presence of rapamycin-only (^Rap^Treg) (Fig 2A).

Previously, others and we described that Treg can lose their stability and start to produce pro-inflammatory cytokines [10, 11]. To analyze the stability of Treg that were expanded in the presence of TNFR2-agonist plus rapamycin, we analyzed their IL-17A and IFNγ producing potential. Under the given stimulatory conditions Treg expanded in the presence of Rap, or Rap+TNFR2 agonist revealed very low levels of IL-17A or IFNγ (Fig 2B).

### TNFR2-agonist promotes efficient ex vivo expansion of lower purity MACS-isolated Treg into a highly stable homogenous Treg population

**Fig 3:**
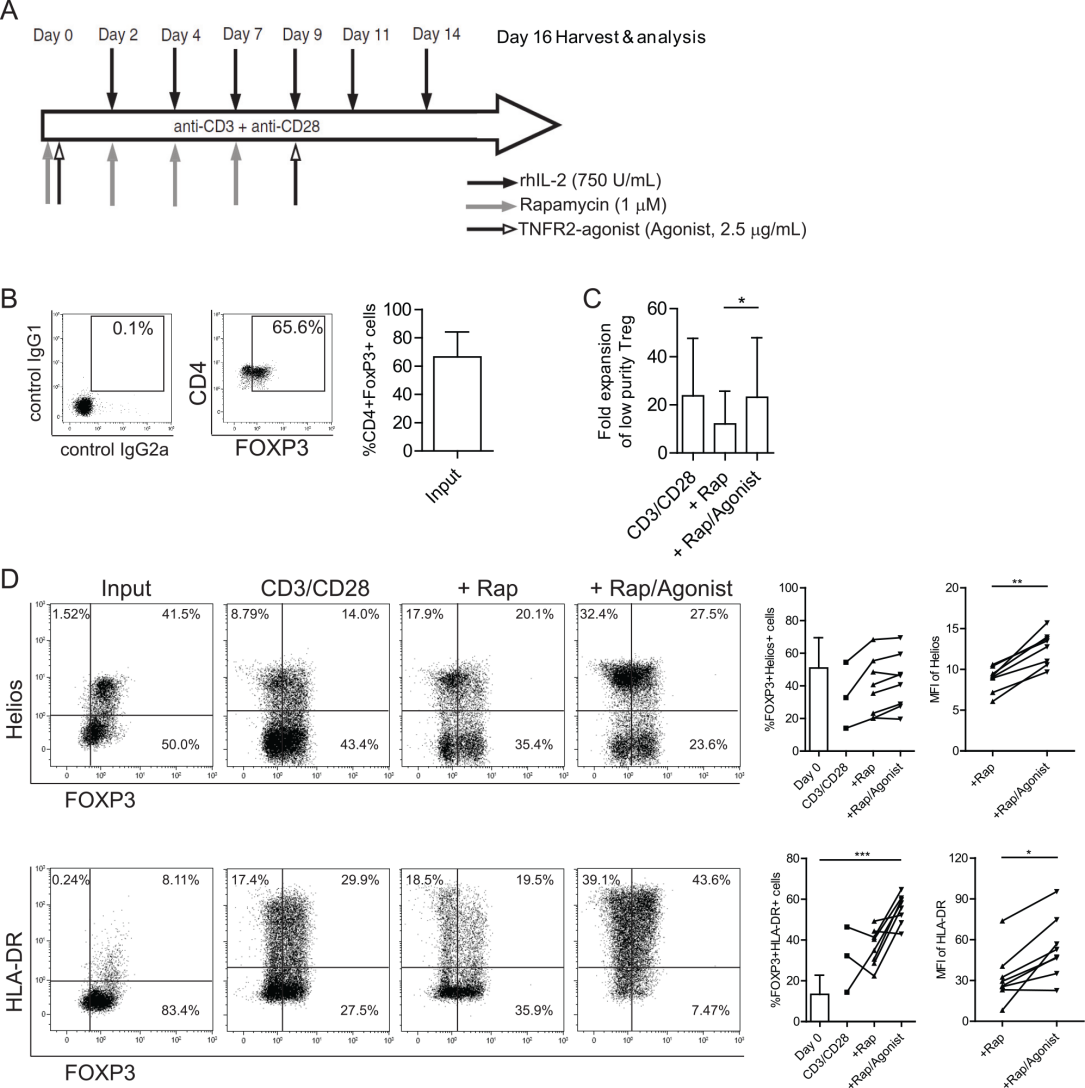
TNFR2-agonist facilitates *ex vivo* expansion of low purity MACS-isolated human Treg. (A) Schematic overview of low purity MACS-isolated Treg expansion strategy as described in Materials and Methods. (B) Dot plots showing a representative FOXP3 expression pattern after MACS isolation of Treg, as shown in the cumulative data graph (N = 10, right panel). (C) Cumulative graph showing fold expansion of low purity Treg that were stimulated under the conditions as indicated on the X-axis (N = 5). A Friedman test was used for comparison of three groups. (D) Flow cytometry of surface expression of HLA-DR, and intracellular expression of FOXP3 and Helios of MACS-isolated Treg before (input) and after expansion under the indicated conditions. Numbers within the quadrant gates show the percentage of positive cells. Cumulative data of %FOXP3+Helios+, %FOXP3+HLA-DR+, the median fluorescence intensity (MFI) of Helios and HLA-DR are shown in the right panel, respectively. Wilcoxon paired t-Test and Kruskal-Wallis test were used for comparison between two and multiple groups, respectively. Asterisks indicate significant differences. Rap: rapamycin; Agonist: TNFR2-agonist.

Then, we further explored the effect of stimulation with the TNFR2-agonist using MACS-isolated, and thus less pure but more easily obtainable, CD4+CD25+ human Treg. We employed a well established Treg expansion protocol [22] that includes anti-CD3/CD28 mAb coated microbead stimulation, high dose rhIL-2 and rapamycin (Fig 3A). To mimic the moderately pure Treg isolated using CliniMACS® which is typically around 40–60% of CD4+CD25^high^ cells [2], we prepared human Treg by using laboratory based MiniMACS® with a modified amount of CD25 beads, thus resulting in a lower purity of Treg as analyzed by the expression of FOXP3 (65.6% ± 18, mean ± SD, Fig 3B). Interestingly, the fold expansion of MACS-isolated Treg in the combined use of TNFR2-agonist plus rapamycin was significantly increased (mean 23-fold expansion) as compared to that of rapamycin-only (mean 12-fold expansion) (Fig 3C). This data indicate that combined use of TNFR2-agonist plus rapamycin could overcome the rapamycin-mediated inhibition of Treg proliferation. Due to the usage of a high amount of rhIL-2 in the expansion protocol, which has shown its critical role in the lineage maintenance of both murine and human Treg [31, 32], FOXP3 expression in the CD3/CD28 group was largely preserved upon stimulation. Interestingly, expression levels of Helios (MFI) and HLA-DR (percentage and MFI) on ^R/T^Treg after expansion were significantly higher than those on ^Rap^Treg; Under both conditions the expression of FOXP3 was preserved up to a similar level (Fig 3D).

**Fig 4:**
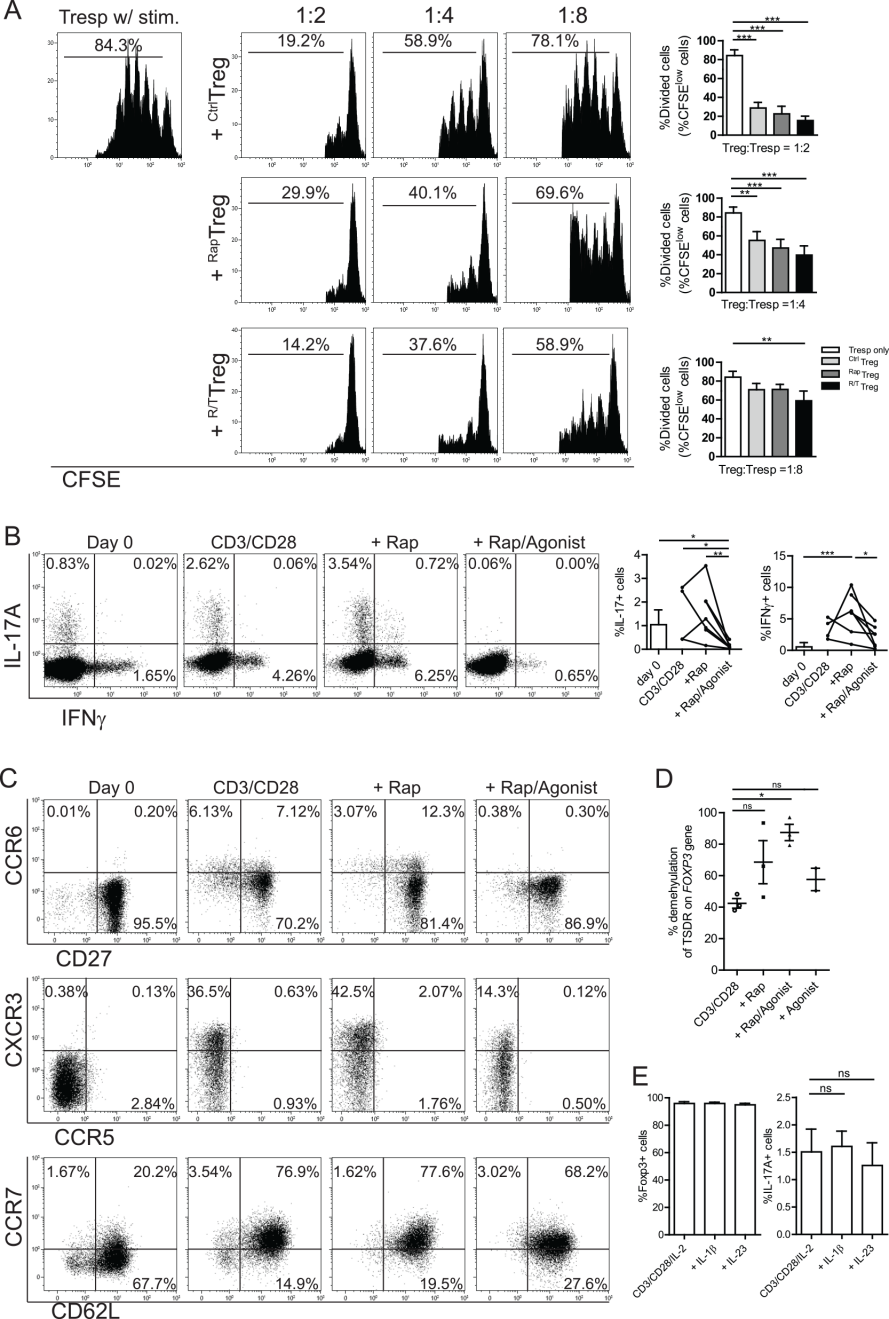
TNFR2-agonist preserves the stability of low purity MACS-isolated Treg during *ex vivo* expansion. Low purity MACS-isolated human Treg were cultured as described under Materials and Methods. Thereafter, the expanded Treg were harvested, washed, and analyzed for their suppressor capacity in a CFSE-based co-culture suppression assay. ^Ctrl^Treg, ^Rap^Treg and ^R/T^Treg represent cells expanded in the absence or presence of rapamycin-only or TNFR2-agonist plus rapamycin, respectively. (A) Histograms showing the inhibition of proliferation of Tresp following the addition of graded doses of Treg. The ratios of Treg:Tresp are indicated on the top. Numbers in the histograms show the percentage of divided cells. Cumulative data (N = 6) are shown in the right panel. (B) Flow cytometry of intracellular IL-17A and IFNγ of Treg at the start of the culture (day 0) and after expansion under the indicated conditions. Dot plots showing representative data of N = 4–7 individuals as shown in the cumulative data graph (right panel). Numbers show the percentage of positive cells. Each line represents Treg derived from a specific donor were expanded under the conditions described on the X-axis. (C) Expression of CXCR3, CCR5, CCR6, CCR7, CD62L and CD27 before (day 0) and after expansion (day 16). Numbers show the percentage of positive cells. (D) Bisulphite sequencing of the TSDR of expanded Treg. Each dot represents a single experiment. (E) Expanded Treg were harvested, rested overnight, and then restimulated with anti-CD3/anti-CD28 beads in a 1:2 ratio of beads to cells, in the absence or presence of IL-1β (50 ng/mL) and IL-23 (50 ng/mL) for 2-days. Exogenous rhIL-2 (200 U/mL) was included in the cell cultures. Thereafter, intracellular production of IL-17A was analyzed using flow cytometry. Cumulative data derived from seven different donors are shown. A Friedman plus Dunns post hoc test (A, D, and E) or Kruskal-Wallis plus Dunns post hoc test (B) were used for comparison among multiple groups. Asterisks indicate significant differences. Rap: rapamycin; Agonist: TNFR2-agonist.

Regarding the suppressor capacity of ^R/T^Treg: these were superior to ^Rap^Treg, as at a lower Treg:Tresp ratio (1:8), only ^R/T^Treg, but not ^Rap^Treg, could significantly inhibit the proliferation of responder cells (Fig 4A). In addition, the TNFR2-agonist plus rapamycin expanded Treg hardly showed the potential to produce IL-17A and IFNγ (Fig 4B), indicating that ^R/T^Treg are more stable than ^Rap^Treg. Expansion of low purity MACS-isolated Treg in the presence of TNFR2-agonist plus rapamycin resulted in a homogenous Treg population that expressed CD62L, CCR7, and CD27, lacked expression of CCR5 and CCR6, while a limited percentage of cells expressed CXCR3 (Fig 4C). This homogenous Treg phenotype and the high suppressive capacity were comparable to the results obtained with high purity FACS-sorted Treg, suggesting that this protocol is of interest for clinical grade *ex vivo* expansion of low purity, but easily obtainable Treg.

As Treg stability and suppressor function critically depends on the stable expression of FOXP3, which in turn depends on hypo-methylation of a CpG rich region in the *FOXP3* gene, called the TSDR, we hypothesized that stimulation by TNFR2-agonist plus rapamycin of Treg promotes demethylation of the TSDR. To test this, low purity MACS-isolated Treg were expanded according to the protocol mentioned and the TSDR demethylation status was analyzed using bi-sulphite sequencing. The significant increase of TSDR demethylation was only observed in TNFR2-agonist plus rapamycin expanded cells, but not in case of rapamycin or TNFR2-agonist only group (Fig 4D). This TNFR2-agonist plus rapamycin induced hypo-methylation of the *FOXP3* gene likely explains the increased suppressor capacity and high stability of Treg population expanded under these conditions.

Previously, we showed that stimulation of human Treg in a pro-inflammatory environment enhances the IL-17A producing potential of Treg [10]. Having established that Treg expansion in the presence of TNFR2-agonist plus rapamycin results in increased stability of the Treg population, we questioned whether re-stimulation of these cells in a pro-inflammatory environment could break the stability and promote the IL-17A producing potential. To examine this, low purity MACS-isolated Treg were expanded according to the protocols mentioned, and the resultant Treg were subsequently re-stimulated with anti-CD3/CD28 beads and rhIL-2 in the absence or presence of the pro-inflammatory cytokines IL-1β or IL-23. Re-stimulation of these expanded Treg in the presence of IL-1β or IL-23 neither led to the loss of FOXP3 expression, nor the increase in IL-17A producing potential (Fig 4E), further stressing the stability of these expanded Treg.

In conclusion, 16 days expansion of low purity MACS-isolated Treg in the presence of TNFR2-agonist and rapamycin results in a highly pure, homogenous and very stable Treg population.

### TNFR2-agonist plus rapamycin expanded Treg inhibit inflammation in a humanised mouse model

**Fig 5:**
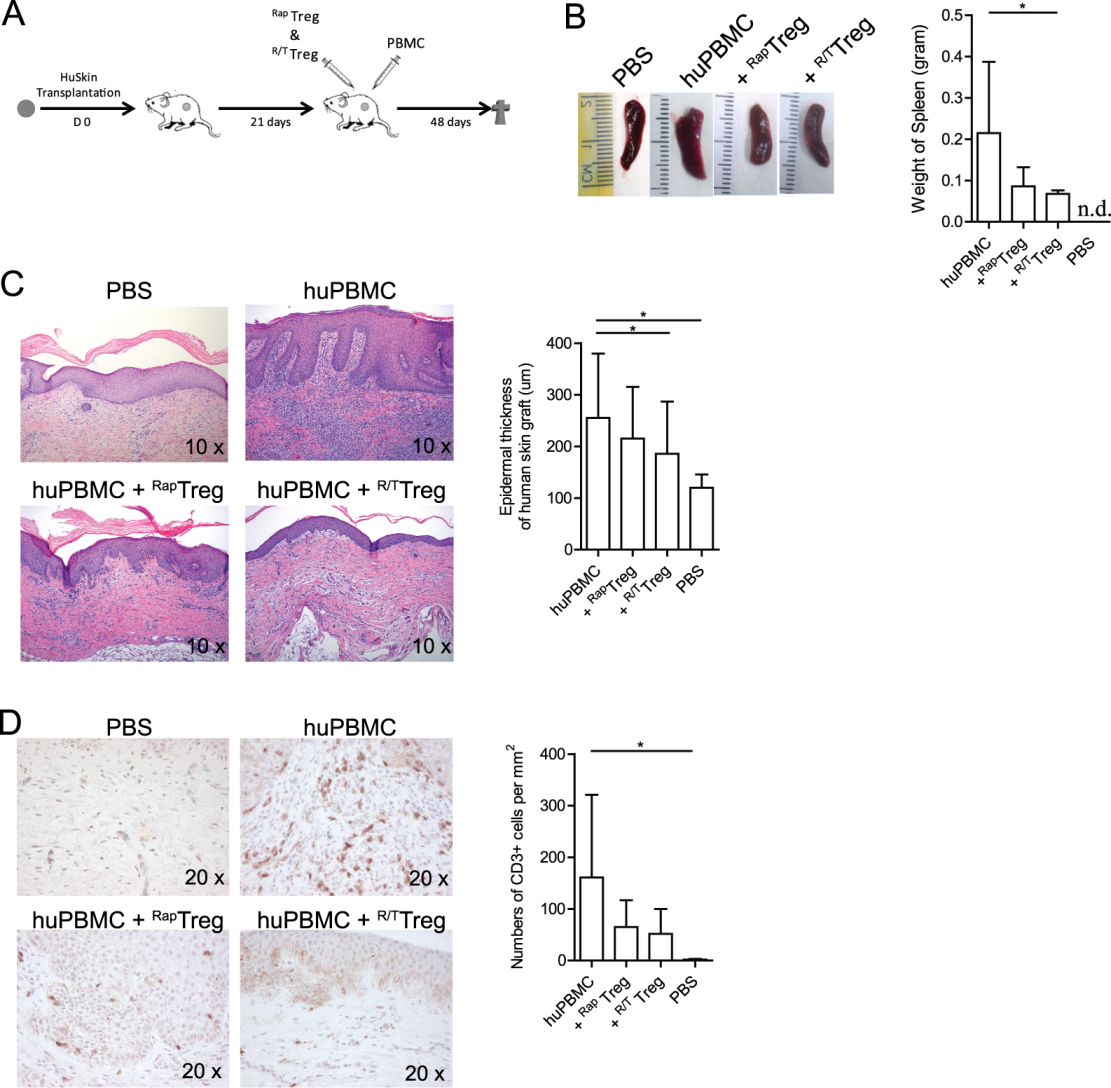
TNFR2-agonist plus rapamycin expanded Treg inhibit inflammation in a humanised mouse model. (A) Schematic overview of the humanised skin inflammation mouse model used. In brief, SCID mice were transplanted with a human skin graft, 21 days after engraftment, PBS (as a control), or allogeneic human PBMC (huPBMC) only or huPBMC plus Treg of interest (at a ratio of 1:1) were injected intra peritoneally. 26 days later the animals were sacrificed to analyze the mouse spleen and human skin grafts. ^Rap^Treg and ^R/T^Treg refer to low purity MACS-isolated Treg expanded for 16-days in the presence of rapamycin-only or TNFR2-agonist plus rapamycin, respectively. (B) Representative photographs of spleens derived from mice infused with PBS, huPBMC only, or huPBMC plus Treg of interest, 21 days after the skin transplantation. Cumulative data showing the weight of spleens derived from N = 4 mice (right panel; n.d. = not determined). (C) Representative photographs showing histology (HE staining) of human skin grafts. Left panel: 10 x magnification. Right panel shows the epidermal thickness (μm) of human skin grafts following infusion of PBS, huPBMC, huPBMC plus ^Rap^Treg or huPBMC plus ^R/T^Treg. Mean ± SD, N = 6. (D) Immunohistochemistry of human CD3+ (brown) T cell infiltration in the human dermis. A representative photograph of N = 4 as presented in the cumulative data graph is shown (right panel; Mean ± SD). 20 x magnification. Wilcoxon paried t Test was used to compare the group of mice infused with huPBMC only with other groups of mice infused with huPBMC plus Treg of interest. Asterisks indicate significant differences.

Next, using a pre-clinical humanised skin inflammation mouse model, we sought to establish whether TNFR2-agonist plus rapamycin expanded low purity MACS-isolated Treg could suppress inflammation *in vivo*. To this end, SCID mice were transplanted with a human skin graft, whereupon 21 days after engraftment of the human skin, PBS (as a control) or allogeneic human PBMC (huPBMC) were injected intra peritoneally. Typically, the latter results in a strong inflammatory response of the human skin 3 weeks after infusion, which is characterized by thickening of the epidermis and influx of human lymphocytes [28]. Systemic repopulation of human lymphocytes was observed as indicated by the increased size of mouse spleen (Fig 5B). Both TNFR2-agonist plus rapamycin expanded Treg and rapamycin-only expanded Treg were suppressive, as indicated by a reduction of epidermal thickening (Fig 5C) and human T cell numbers in the dermis of the grafted skin (Fig 5D); under the given conditions levels of in vivo inhibition were not significantly different between the two types of Treg.

## Discussion

An important issue for Treg-based immunotherapy is to maintain stability and suppressive function of Treg during and after *ex vivo* expansion and following their transfer into patients. Although clinical grade high purity Treg isolation by GMP flow cytometry is available in a few medical centres worldwide, most clinic centres use GMP qualified magnetic bead based isolation techniques that result in limited Treg purity. Hence, in this study we focused on optimizing an *ex vivo* Treg expansion protocol that produces high numbers of stable potent human Treg starting with low purity magnetic bead isolated Treg. We found that the combined use of TNFR2-agonist and rapamycin promotes Treg proliferation rates, enhances TSDR demethylation and increases both Treg stability and function *in vitro*. Low purity Treg expanded in the presence of TNFR2-agonist plus rapamycin suppressed *in vivo* inflammation in a humanized mouse model.

TNFα has both pro-inflammatory and anti-inflammatory effects. It binds to two structurally related but functionally distinct receptors TNFR1 and TNFR2. In general, TNFR1 is responsible for TNFα-mediated cell apoptosis, and TNFR2 for any function related to T cell survival. In contrast to the ubiquitous expression of TNFR1, TNFR2 expression is more limited to myeloid and lymphoid cell lineages [18]. Interestingly, human Treg, as opposed to CD25- Tconv cells, constitutively express high levels of TNFR2, and TNFR2+ Treg reveal the most potent suppressive capacity [20, 24]. The effect of TNFα on Treg suppressor function remains controversial. Some groups reported that TNFα/TNFR2 signalling inhibits human Treg suppressive function [33, 34], other groups found that TNFα increases FOXP3 expression and suppressive activity, and that TNFR2 is crucial for sustaining FOXP3 expression and maintaining the stability of murine Treg in an inflammatory environment [20]. It should be noted that a more recent study revealed that the nature of the TNFR2 antibodies used in these studies was likely different (agonistic versus antagonistic) [22]. In this study, we found that stimulation of human Treg with a TNFR2-agonist antibody preserved a stable Treg phenotype and function after *ex vivo* expansion. It is known that TNFα induces a TNFR2/NFκB dependent pathway in human Treg, and that an increase in NFκB activity promotes FOXP3 expression [33]. Using TNFR2-agonist only was enough to prevent the loss of FOXP3 expression during *ex vivo* expansion, whereas sustained hypo-methylation of TSDR required both rapamycin and TNFR2-agonist, suggesting that stabilization of FOXP3 expression requires both mTOR and NFκB signal pathways. One of the major concerns in Treg therapy is the plasticity of Treg, and the hypomethylation of TSDR is well correlated to the loss of Treg stability [35]. *In vitro* re-stimulation of TNFR2-agonist plus rapamycin expanded Treg led to neither the loss of FOXP3 expression, nor the enhancement of IL-17A production, especially under pro-inflammatory conditions, suggesting a well preserved Treg stability. This observation appears to be supported by our *in vivo* data acquired from a humanized mouse model for skin inflammation.

The clinical application of Treg based adoptive therapy in transplantation and autoimmunity is hampered by the paucity of peripheral Treg numbers, purity of clinical grade isolated Treg and stability and function of *ex vivo* expanded Treg. Current protocols used for Treg *ex vivo* expansion commonly use anti-CD3 and anti-CD28 mAb stimulation in the presence of rhIL-2 [2–8]. This treatment alone typically results in a heterogeneous Treg population revealing inadvertent pro-inflammatory (like IL-17A, IFNγ) cytokine producing potential [9–11]. Moreover, Treg isolation using the GMP CliniMACS® system leads to a moderately pure Treg population, with around 40–60% CD4+CD25^high^ cells [2]. Considering the intrinsic reduced ability of Treg to proliferate *in vitro*, “contaminating” non-Treg cells might overgrow Treg during *ex vivo* expansion. One solution is to include rapamycin, an effective inhibitor of effector T cells, in the expansion culture. However, addition of rapamycin generally leads to lower overall cell yields [36]. Consistent with the report that TNFR2/NFκB pathways stimulate human Treg proliferation [33], combined use of TNFR2-agonist and rapamycin resulted in efficient Treg proliferation. These fully expanded Treg were even more suppressive than cells expanded in the presence of rapamycin-only. Previous studies showed that the use of rapamycin leads to the inhibition of IL-17A and IFNγ production [37]. Intriguingly, combined use of TNFR2-agonist and rapamycin further prevented IL-17A and IFNγ production, as compared to rapamycin-only treatment. Moreover, the percentage of CCR6 positive cells, a marker that identifies IL-17-producing cells derived from human Treg [10], was also low following the treatment with TNFR2-agonist plus rapamycin. A recent study showed that TNFR2 knock out CD4+ T cells had increased expression of RORγt and IL-17 production, which was dependent on the impairment of TNFR2-mediated activation of NFκB [38]. We speculate that a similar process of regulation may exist in human Treg where TNFR2/NFκB signalling might act as a double-edged sword to enhance FOXP3, but inhibit RORγt expression, thus contributing to the stabilization of Treg.

Previous studies indicated that TNFR2 is more densely expressed on human CD45RA-activated Treg [33]. Therefore, stimulation with a TNFR2-agonist might mainly induce the proliferation of a memory Treg subset. Indeed, one of the most notable surface markers that was up-regulated by stimulation in the presence of TNFR2-agonist was HLA-DR, which identifies an effector Treg subset that exhibits higher FOXP3 expression and more potent suppression [30]. HLA-DR positive effector Treg were reported to be more sensitive to apoptosis than HLA-DR negative Treg [39]. However, in this study, re-stimulation of ^R/T^Treg, which expressed a high level of HLA-DR, had a similar cell viability as ^Ctrl^Treg that express little HLA-DR. Analysis of chemokine receptors expression showed that stimulation of the TNFR2-agonist led to reduced expression of the chemokine receptors CXCR3 and CCR6, which are linked to Th1 and Th17 like cells, respectively. An implication of the lack of CXCR3 and CCR6 on the expanded Treg might suggest that these cells upon infusion fail to migrate to sites of Th1 and Th17 responses. However, all TNFR2-agonist expanded Treg showed CD62L expression, which might favour their trafficking to secondary lymphoid organs, where they might further expand and receive instruction with regard to tissue homing capacity [27].

In conclusion, we demonstrate the potential of additional TNFR2-agonist stimulation for *ex vivo* expansion of low purity Treg. Expansion of low purity MACS-isolated human Treg in the presence of TNFR2-agonist and rapamycin results in a stable homogenous FOXP3+Helios+ Treg population that reveals potent suppressor potential both *in vitro* and *in vivo*, in a preclinical humanized mouse model. Our findings further emphasize that expansion of bead-isolated Treg requires rapamycin for achieving a functional and stable Treg cell product. But, the selection of an additional agent like TNFR2-agonist can overcome the rapamycin-mediated inhibition of Treg proliferation, and even further stabilize the Treg phenotype based on the demethylation status of TSDR region. It is thus of great interest to consider the combined use of TNFR2-agonist and rapamycin for stable *ex vivo* Treg expansion for clinical application.

## Supporting Information

S1 Fig TNFR2-agonist down regulates the expression of CCR6 and CXCR3, while hardly affecting CCR7 expression. Flow cytometry of FACS-sorted Treg after cell expansion under the indicated conditions. Dot plots showing surface expression of CXCR3, CCR5, CCR6, CCR7, CD62L and CD27 at day 7 of the cell cultures. Numbers within the quadrant gates show the percentages of positive cells. Data derived from two different donors are shown. Rap: rapamycin; Agonist: TNFR2-agonist.
(EPS)
